# Supplementary material for: Spatially expandable fiber-based probes as a multifunctional deep brain interface
Source: Nat Commun. 2020 Nov 30;11:6115. doi: 10.1038/s41467-020-19946-9 (PMC7704647; doi:10.1038/s41467-020-19946-9)
Supplement: Supplementary file 3 — Reporting Summary [file 41467_2020_19946_MOESM3_ESM.pdf]

nature research

Corresponding author(s): Xiaoting Jia

Last updated by author(s): Sep 24, 2020

Reporting Summary

Nature Research wishes to improve the reproducibility of the work that we publish. This form provides structure for consistency and transparency in reporting. For further information on Nature Research policies, seeAuthors & Referees and theEditorial Policy Checklist .

Statistics

For all statistical analyses, confirm that the following items are present in the figure legend, table legend, main text, or Methods section.

n/a	Confirmed	
☐	☒	The exact sample size (n) for each experimental group/condition, given as a discrete number and unit of measurement
☐	☒	A statement on whether measurements were taken from distinct samples or whether the same sample was measured repeatedly
☐	☒	The statistical test(s) used AND whether they are one- or two-sided<br>*Only common tests should be described solely by name; describe more complex techniques in the Methods section.*
☐	☒	A description of all covariates tested
☐	☒	A description of any assumptions or corrections, such as tests of normality and adjustment for multiple comparisons
☐	☒	A full description of the statistical parameters including central tendency (e.g. means) or other basic estimates (e.g. regression coefficient) AND variation (e.g. standard deviation) or associated estimates of uncertainty (e.g. confidence intervals)
☐	☒	For null hypothesis testing, the test statistic (e.g. F, t, r) with confidence intervals, effect sizes, degrees of freedom and P value noted<br>*Give P values as exact values whenever suitable.*
☒	☐	For Bayesian analysis, information on the choice of priors and Markov chain Monte Carlo settings
☒	☐	For hierarchical and complex designs, identification of the appropriate level for tests and full reporting of outcomes
☒	☐	Estimates of effect sizes (e.g. Cohen's d, Pearson's r), indicating how they were calculated

Our web collection on statistics for biologists contains articles on many of the points above.

Software and code

Policy information about availability of computer code

Data collection	All the software used in the data collection has bee mentioned in the manuscript. We used Acqknowledge 5.0 from BIOPAC for acquiring and analyzing electrographic recordings.
Data analysis	Data analysis was performed with customized scripts in Matlab (The Mathworks).

For manuscripts utilizing custom algorithms or software that are central to the research but not yet described in published literature, software must be made available to editors/reviewers. We strongly encourage code deposition in a community repository (e.g. GitHub). See the Nature Research guidelines for submitting code & software for further information.

Data

Policy information about availability of data

All manuscripts must include a data availability statement. This statement should provide the following information, where applicable:

- Accession codes, unique identifiers, or web links for publicly available datasets
- A list of figures that have associated raw data
- A description of any restrictions on data availability

All figures have associated raw data. The data that support the findings of this study are available from the corresponding author upon reasonable request.All Matlab codes are available upon reasonable request to the corresponding author.

Field-specific reporting

Please select the one below that is the best fit for your research. If you are not sure, read the appropriate sections before making your selection.

Life sciences study design

All studies must disclose on these points even when the disclosure is negative.

Sample size	For EIS and stiffness measurements, n=3. For recording in wild type mice, n=5. For recording in Thy1-ChR2-YFP, n=3 (depth dependent fiber probes) and n=5 (spatially expandable fiber probes). For seizure studies, n=3 (straight implant), n=4 (angled implant), and n=3 (spatially expandable fiber probes).
Data exclusions	No data were excluded for analysis.
Replication	The study was repeated in multiple animals (n≥3) which indicate the replication of our work.
Randomization	By the nature of this experiment, randomization is not relevant.
Blinding	By the nature of this experiment, blinding is not relevant.

Reporting for specific materials, systems and methods

We require information from authors about some types of materials, experimental systems and methods used in many studies. Here, indicate whether each material, system or method listed is relevant to your study. If you are not sure if a list item applies to your research, read the appropriate section before selecting a response.

Materials & experimental systems

n/a	Involved in the study	
☐	☒	Antibodies
☒	☐	Eukaryotic cell lines
☒	☐	Palaeontology
☐	☒	Animals and other organisms
☒	☐	Human research participants
☒	☐	Clinical data

Methods

n/a	Involved in the study	
☒	☐	ChIP-seq
☒	☐	Flow cytometry
☒	☐	MRI-based neuroimaging

Antibodies

Antibodies used	GFAP, Iba1, NeuN.
Validation	The choices of these antibodies were based on previously published work.

Animals and other organisms

Policy information about studies involving animals; ARRIVE guidelines recommended for reporting animal research

Laboratory animals	Wild type mice, male, 5 weeks - 2 months old; Thy1-ChR2-YFP, male and female, 9 weeks - 5 months old.
Wild animals	The study did not involve wild animals.
Field-collected samples	This study did not involve the samples collected in the filed.
Ethics oversight	All animal procedures were approved by Virginia Tech Institutional Animal Care and Use Committee and Institutional Biosafety Committee and were carried out in accordance with the National Institutes of Health Guide for the Care and Use of Laboratory Animals.

Note that full information on the approval of the study protocol must also be provided in the manuscript.
